# Molecular Reconstruction of the Diet in Human Stool Samples

**DOI:** 10.1128/mSystems.00634-19

**Published:** 2019-11-05

**Authors:** Frank Maixner

**Affiliations:** a Eurac Research - Institute for Mummy Studies, Bolzano, Italy

**Keywords:** DNA barcodes, diet, microbiome

## Abstract

Understanding dietary effects on the gut microbial composition is one of the key questions in human microbiome research. It is highly important to have reliable dietary data on the stool samples to unambiguously link the microbiome composition to food intake. Often, however, self-reported diet surveys have low accuracy and can be misleading. Thereby, additional molecular biology-based methods could help to revise the diet composition. The article by Reese et al. [A. T. Reese, T. R. Kartzinel, B. L. Petrone, P. J. Turnbaugh, et al., mSystems 4(5):e00458-19, 2019, https://doi.org/10.1128/mSystems.00458-19] in a recent issue of *mSystems* describes a DNA metabarcoding strategy targeting chloroplast DNA markers in stool samples from 11 human subjects consuming both controlled and freely selected diets. The aim of this study was to evaluate the efficiency of this molecular method in detecting plant remains in the sample compared to the written dietary records. This study displays an important first step in implementing molecular dietary reconstructions in stool microbiome studies which will finally help to increase the accuracy of dietary metadata.

## Abstract (precis)

Understanding dietary effects on the gut microbial composition is one of the key questions in human microbiome research. It is highly important to have reliable dietary data on the stool samples to unambiguously link the microbiome composition to food intake. Often, however, self-reported diet surveys have low accuracy and can be misleading. Thereby, additional molecular biology-based methods could help to revise the diet composition. The article by Reese et al. [A. T. Reese, T. R.

## COMMENTARY

Diet is an important external factor influencing the composition of the human gut microbiome. Various studies have shown the effects of dietary changes on gut microbes which can subsequently impact human physiology (1). The experimental setup of these studies often included a major change in individual macronutrients (e.g., high-fiber diet) (2). However, since the macronutrients are rarely consumed in isolation, those human gut microbiome studies must in addition rely on self-reported human diet surveys. This brings human error into play, resulting in inaccurate dietary surveys due to biases associated with human memory (3). Therefore, it would be of utmost importance to include in future diet-microbiome studies further molecular methods that allow an independent dietary reconstruction of the analyzed stool samples.

The idea to molecularly reconstruct diet in stool samples is not new and has already been applied in the field of ecology and ancient DNA research. DNA barcodes targeting both plant (trnL/UAA intron, large subunit of the RuBisCO) (4, 5) and animal (16S ribosomal DNA [rDNA], 12S rDNA, cytochrome *b*) (4, 6, 7) plastid regions have previously been used to reconstruct the diet, e.g., of wild herbivore (8) and omnivore (9) animals. Furthermore, metabarcodes have been used to analyze the flora and fauna of past and present ecosystems (10, 11). Recently, the last meal of the Iceman, a 5,300-year-old European glacier mummy, has been reconstructed from an ancient stool sample by using an interdisciplinary approach (7). The authors combined classical microscopy with various -omics strategies (genomics, proteomics, lipidomics, metabolomics) including DNA metabarcoding to unambiguously identify the plant and animal remains in the Iceman’s diet.

Resse and colleagues (12) applied in their paper in a recent issue of *mSystems* a DNA metabarcoding approach targeting the plant marker trnL/UAA intron to characterize the plant diet in stool samples collected from 11 human subjects consuming both controlled and freely selected diets. The study evaluated the efficiency of this molecular method in detecting plant remains in human stool samples by comparing the written dietary records of the patients to the molecular results. The authors observed a high detection rate of plant taxa in the molecular survey compared to the written diet records from controlled settings. Importantly, DNA metabarcoding could distinguish between prepared diets with defined sets of plant ingredients and noninterventional diet compositions. Beside these first promising results, the authors are fully aware of the current limitations of the applied DNA metabarcoding approach. First, the PCR amplification success rate ranged between 50% and 70%. Thus, not all samples could be analyzed. Second, metabarcoding detects plant traces in freely eaten diets that may not reflect the main plant diet but rather ingredients possibly included in processed foods. Third, the metabarcoding approach is always as good as the quality and completeness of the comparative data set. Especially since one future goal should be to apply the approach to more geographically and culturally diverse human populations, I would assume that the comparative marker data sets must be adapted to geographical dietary habits, maybe to plant taxa not yet present in the NCBI sequence depository.

The work by Reese et al. (12) shows that DNA-based dietary analysis holds promise for tracking human plant diet molecularly. Future studies can be extended to other DNA barcodes to also characterize the animal diet in omnivorous studies. As a long-term goal, PCR-independent metagenomic dietary analysis in stool samples could become a routine method that will allow in combination with other -omics technologies (e.g., proteomics, lipidomics) the full reconstruction of human diets. Dietary information obtained by that means combined with gut microbiome compositional data will open new avenues in the understanding of the effects of dietary changes to microbial structure and subsequently to human physiology.
